# Functional connectivity of brain networks during semantic processing in older adults

**DOI:** 10.3389/fnagi.2022.814882

**Published:** 2022-10-19

**Authors:** Amanda Garcia, Ronald A. Cohen, Eric C. Porges, John B. Williamson, Adam J. Woods

**Affiliations:** Center for Cognitive Aging and Memory, McKnight Brain Institute, Departments of Clinical and Health Psychology and Psychiatry, University of Florida, Gainesville, FL, United States

**Keywords:** functional connectivity, fMRI, semantics, seed-to-voxel, hubs, preservation of function, aging

## Abstract

The neural systems underlying semantic processing have been characterized with functional neuroimaging in young adults. Whether the integrity of these systems degrade with advanced age remains unresolved. The current study examined functional connectivity during abstract and concrete word processing. Thirty-eight adults, aged 55–91, engaged in semantic association decision tasks during a mixed event-related block functional magnetic resonance imaging (fMRI) paradigm. During the semantic trials, the task required participants to make a judgment as to whether pairs were semantically associated. During the rhyme trials, the task required participants to determine if non-word pairs rhymed. Seeds were placed in putative semantic hubs of the left anterior middle temporal gyrus (aMTG) and the angular gyrus (AG), and also in the left inferior frontal gyrus (IFG), an area considered important for semantic control. Greater connectivity between aMTG, AG, and IFG and multiple cortical areas occurred during semantic processing. Connectivity from the three seeds differed during semantic processing: the left AG and aMTG were strongly connected with frontal, parietal, and occipital areas bilaterally, whereas the IFG was most strongly connected with other frontal cortical areas and the AG in the ipsilateral left hemisphere. Notably, the strength and extent of connectivity differed for abstract and concrete semantic processing; connectivity from the left aMTG and AG to bilateral cortical areas was greater during abstract processing, whereas IFG connectivity with left cortical areas was greater during concrete processing. With advanced age, greater connectivity occurred only between the left AG and supramarginal gyrus during the processing of concrete word-pairs, but not abstract word-pairs. Among older adults, robust functional connectivity of the aMTG, AG, and IFG to widely distributed bilateral cortical areas occurs during abstract and concrete semantic processing in a manner consistent with reports from past studies of young adults. There was not a significant degradation of functional connectivity during semantic processing between the ages of 55 and 85 years. As the study focused on semantic functioning in older adults, a comparison group of young adults was not included, limiting generalizability. Future longitudinal neuroimaging studies that compare functional connectivity of young and older adults under different semantic demands will be valuable.

## Introduction

The elements, organization, and processes of semantic memory have been approached from multiple theoretical and research perspectives. Early linguistically based theories tended to consider semantic memory as consisting of a set of symbolic representations based on their linguistic collocation or their associative relationships to one another, determinable through latent semantic analyses (Meteyard et al., 2012). Models developed from this perspective considered semantic representations of words and symbols as distinct from associative representations to which they refer. Early cognitive theories extended linguistic-based models of semantics, incorporating constructs from studies of memory processing and storage. Semantic associations are stored like other types of information in a single long-term memory (LTM), though little attention was given to the location of LTM in the brain, or how memory was physically stored. Advances in computational approaches to cognitive science led to most researchers to conclude that LTM was not a monolithic storage compartment, but that rather that it consisted of large associative networks, such as proposed in Anderson’s human associative memory theory (Anderson and Bower, 1973).

Subsequent efforts were directed at harmonizing principles from associative memory theories with emerging evidence regarding the operating principles underlying brain structure and function. Connectionist models considered semantic associative memory to consist of associative networks widely distributed across the cortex. For example, the parallel distributed processing (PDP) framework considered associative memory be a manifestation of interactions among elementary associative information derived from sensory input (Rumelhart and McClelland, 1986; McClelland and Rumelhart, 1989). Symbolic information was considered to be distributed across neural systems in a manner similar to that of other types of associations, but on a symbolic gradient (Smolensky et al., 2014). Within models developed from this theoretical perspective, semantic memory is considered to “embodied,” as it is organismic byproduct of elementary attributes of human’s sensory experience (Gallese and Lakoff, 2005). Semantic embodiment assumes that cognition is constrained by biological realities of brain structure and function (Kumar, 2021; Kumar et al., 2022).

Clinical neuropsychological and cognitive neuroscience research have provided evidence that semantic associative memory is embodied and widely distributed across the cortex. It has also supported the existence of specialized cortical systems that process linguistic information and link symbolic representations with embodied semantic associations. Studies of semantic dementia provided evidence that semantic memory is widely distributed across cortical areas. Patients with this neurodegenerative disease experience severe impairments in their ability to derive meaning from language (Warrington, 1975; Mesulam et al., 2009; Deleon and Miller, 2018). With disease progression, widespread cortical atrophy occurs, not limited to one isolated cortical region, with semantic impairments worsening as cortical atrophy increases (Chan et al., 2001; Gainotti, 2017; Gazzina et al., 2017; Macoir et al., 2017; Mann and Snowden, 2017; Deleon and Miller, 2018). However, atrophy of anterior temporal lobes, including the temporal poles, is particularly prominent in semantic dementia, suggesting that this cortical area may be important for semantic processing. Studies of the effects of stroke provide evidence for the role of other specialized cortical areas involved in semantic processing (Sharp et al., 2004a). Significant semantic impairments occur from infarctions of more posterior temporal areas and the angular gyrus of the parietal cortex. The supramarginal gyrus and precuneus have also been implicated, though less consistently (Hillis and Caramazza, 1995; Hillis and Caramzza, 1995; Cloutman et al., 2009). Lesions affecting the prefrontal cortex also affect semantic processes, particularly when semantic control is necessary (Sharp et al., 2004b).

Accordingly, there is evidence outside of clinical populations that multiple cortical areas contribute to semantic functioning, though the extent to which specific areas are essential has been the subject of considerable debate. Functional neuroimaging studies have demonstrated the involvement of specific neural systems in semantic processing among people without brain disorders. Meta-analyses, including an analysis of 120 functional neuroimaging studies conducted prior to 2009, have provided evidence that modality-specific cortical regions, as well as multi-modal or amodal convergence zones are active during semantic processing (Binder et al., 2009; McNorgan, 2012). For example, temporal and frontal cortical convergence areas are responsive to multiple information types, integrating associative information that may have originally been generated by processing across sensory modalities. Neuroimaging findings have been very consistent in showing the involvement of the anterior temporal cortex, including the temporal pole in semantic processing (Jackson et al., 2016, 2018, 2021; Jackson, 2021). However, more posterior perisylvian and parietal cortical areas (e.g., angular gyrus) often are activated on functional magnetic resonance imaging (fMRI) paradigms involving semantic processing (Binder et al., 2009). Activation of the angular gyrus occurs across different semantic paradigms, sensory modalities and word class (e.g., concrete vs. abstract) (Binder et al., 2009), whereas lexical decision-making tends to produce much greater modality-specific activation on fMRI (Bonner et al., 2013). The AG and other perisylvian convergence zones have high synaptic density and connectivity with modality-specific association areas.(Binder et al., 2009; Bonner et al., 2013).

Several conceptual frameworks have been proposed to characterize the neural systems underlying semantic processing and roles played by specific cortical areas. Patterson and colleagues proposed a “hub-and-spoke” model in which the anterior temporal cortex was considered to serve as the primary semantic “hub” (Patterson et al., 2007; Jefferies, 2013; Ralph et al., 2017; Jackson et al., 2018). In other words, a convergence zone where associative information from multiple sensory modalities (i.e., spokes) is integrated to form unified amodal associations (i.e., concepts). Is there only one semantic hub or association area? Reilly et al. (2016) hypothesized that semantic processing depends on higher- and lower-order hubs working in tandem to integrate associative information distributed across more extensive cortical areas. Thus, semantic memory is subserved by a series of hubs that re-engage sensorimotor associations during the processing of object concepts. The anterior temporal area was posited as a high-order semantic hub, as it integrates semantic associative information from more elementary associations derived from the sensory modalities. The angular gyrus and middle temporal gyrus were posited to make up a low-order semantic hub that serves to integrate symbolic and embodied sensory-derived associative information (Jouen et al., 2015). These low-order hubs have massive reciprocal connectivity with sensorimotor regions, such that they are well suited for heteromodal feature binding (Bonner et al., 2013). The angular gyrus is important for creating combinatorial semantic relationships between congruent concepts (Jefferies et al., 2010; Bonner et al., 2013; Graves et al., 2013; Price et al., 2015). Structural connectivity studies employing tractography show that the angular gyrus and temporal pole are strongly connected. These areas also tend to activate on both verbal and non-verbal tasks such as reading a sentence describing an event and viewing a picture of the same event, suggesting that the function of the angular gyrus is not limited to language processing (Jouen et al., 2015). Ultimately, there is considerable evidence that more than one cortical area plays a role in semantic processing; the extent to which specific areas are involved depends on semantic task demands (Binder et al., 2009; Bonner et al., 2013; Reilly et al., 2016). Conceptual frameworks (e.g., Multiple Demand Networks) that posit that two or more cortical areas play key roles in semantic processes depending on semantic demands account for the differences in the intensity of activation and in functional connectivity based on the type of semantic operations that are needed on particular tasks.

How these hypothesized cortical areas interact during semantic processing remains unresolved. Does the relative engagement of cortical areas depend on semantic demands, such as processing concrete vs. abstract words? Functional neuroimaging studies have provided some insights into this question. Greater left hemisphere activation during the processing of abstract words has been found on fMRI, possibly reflecting the need for verbal mediation of abstract concepts (Noppeney and Price, 2004; Binder et al., 2005; Sabsevitz et al., 2005). Alternatively, concrete words may activate more extensive and cortical association areas given that they are more strongly embodied and linked to the physical attributes of past sensory experience (Pexman et al., 2007). Abstract semantic processing has also been shown to produce activation of the ventral prefrontal cortex, which may reflect increased executive control demand of abstract processing (Binder et al., 2009). The ventral prefrontal cortex likely serves as a semantic control network that works in tandem with hubs of the semantic network for abstract concept creation, generating semantic output. When abstract words or concepts are used to facilitate higher-cognitive functions such as planning goal-oriented behavior and problem solving, other prefrontal cortical areas may also be engaged. The extent of prefrontal engagement likely depends on semantic task demands, with greater prefrontal engagement occurring when control of semantic networks is needed. This system would be engaged for complex word or concept selection, when there is ambiguity in the associative information to be processed, when meaning is derived from sentence or situational context, when semantic generation is required, or for inhibition of related near-associations. Abstract word processing is more complex, possibly requiring such engagement.

Prior neuroimaging research has focused primarily on semantic processing among healthy young to middle aged adults. Less is known about the extent to which alterations in the semantic network occur among older adults. Some but not all cognitive functions decline as people reach advanced age. Fluid cognitive functions, including learning efficiency, retrieval from memory, executive-attentional functions, working memory, and processing speed are most susceptible to age-associated decline (Salthouse, 1976, 2000; Salthouse et al., 2000; O’Shea et al., 2018). In contrast, language abilities (e.g., vocabulary, comprehension) are among the crystalized cognitive functions that remain quite stable in older adults without neurodegenerative disease (Ashendorf et al., 2009; Vaughan et al., 2016; O’Shea et al., 2018; Cohen et al., 2019; Stern et al., 2019), though age-associated declines are evident on word generation and naming tasks (Albert et al., 1988; Ramsay et al., 1999; Mackay et al., 2002; Brickman et al., 2005). Semantic functions tend to be preserved, as even the oldest old can derive meaning from language (Eustache et al., 1998; Murphy et al., 2008; White et al., 2012; Vaughan et al., 2016; Zhuang et al., 2016). On the other hand, age-associated structural brain changes occur as people age, including reductions in regional cortical volumes, thickness, and white matter integrity, particularly in pre-frontal cortex, though most cortical areas exhibit some reduction over time (Raz et al., 1997, 1998; Badre et al., 2005). Given that semantic associative memory is distributed across cortical areas, some changes in semantic memory and processing capacity might be expected, particularly when certain task demands exist (i.e., higher order or increased executive demands). Furthermore, reductions in white matter integrity that disrupt anterior-posterior, cross-callosal, and connections between essential hubs could be very detrimental (Sullivan and Pfefferbaum, 2006; Sullivan et al., 2006; Madden et al., 2009a,b). Age-associated reductions in semantic functions may be more evident on tasks that require significant involvement of the pre-frontal cortex for executive and attentional control, and communication between this area and posterior semantic hubs. The extent to which age-associated alterations in blood flow detected during fMRI occur during certain types of semantic processing demands is not yet well understood.

The current study investigated the nature of the semantic system in older adults for both abstract and concrete word processing, using a semantic association paradigm (i.e., judging the relationship between word pairs). Given that abstract words do not have high sensorimotor features and are generally more ambiguous, we hypothesized that during the processing of concrete words, functional connectivity between a hub, secondary association areas, and sensory perceptual areas would be more coherent. Finally, we investigated functional connectivity in the context of age. We predicted that functional connectivity between the hub and other association areas would decrease as a function of age in older adults, but to a lesser extent in concrete words than in abstract words.

## Materials and methods

### Participants

Thirty-eight older adults (22 female; Age = 71.7 ± 10.9 years; Education = 16.3 ± 2.5 years) who had completed a study of successful cognitive aging served as participants. The sample ranged in age from 55 to 85 years. The group consisted of individuals without evidence of neurodegenerative disease (e.g., Alzheimer’s or Parkinson’s disease, Mild Cognitive Impairment [MCI]). Exclusion criteria included a prior diagnosis of neurological or major psychiatric disorder (e.g., schizophrenia) and/or impaired cognitive performance on the Montreal Cognitive Assessment, a brief cognitive screening measure (MoCA ≤ 21). In addition, if participant met inclusion criteria on the MoCA, then a clinical interview was conducted, along with supplementary memory measures, and a clinical dementia rating (CDR) was derived. Adjudication of MCI status was done using this data, and only participants with a CDR = 0 were included in the study. Standard MRI contraindications were a basis for exclusion of potential participants (e.g., claustrophobia, pacemaker, other ferromagnetic body implants). The MoCA cutoff was chosen to optimize the trade-off between sensitivity and specificity of the measure for clinical impairment, which was based on recent evidence that the typical cut-off score of 26 may be too harsh (Waldron-Perrine and Axelrod, 2012). Participants were right-handed (self-report). Participants were not excluded if they spoke languages other than English; however, all participants identified English as their primary language (self-report) and had normal language performance on neuropsychological measures presented in English [Heaton T scores > 30 on the Boston Naming Test (Van Gorp et al., 1986) and Controlled Oral Word Association Test] (Eslinger et al., 1985; Ruff et al., 1996). Notably, an additional six participants were scanned but were excluded as a result of quality control measures (excessive movement or poor behavioral data). Participants provided informed consent, and all study protocols were approved by the University of Florida’s Institutional Review Board.

### Stimuli

Stimuli included a subset of words used in Troche et al. (2014). Ratings from the MRC Psycholinguistic Database were used to classify the words as concrete (Concreteness > 500 on a 100–700 scale) or abstract (Concreteness < 450) (Coltheart and Evans, 1981). Additionally, only words with high familiarity (1 SD above the mean, >587) were used. Pairs were created by extracting associates from Troche et al. (2014) derived from the Florida free association norms (Nelson et al., 2004). To increase variability in responses, additional word-pairs were created from words that were not listed as free associates in the Florida free association norms. These pairs were created by dividing the original Troche word list by level of concreteness (i.e., abstract/concrete) and then randomly assigning each word to a pair within its concreteness level. Level of similarity and association for each word pair was then normed with a series of surveys via Amazon Mechanical Turk (Total *N* = 232). These surveys queried degree of association on a seven-point Likert scale. Word pairs were considered associated if the mean rating was greater than three and “no association” if the mean rating was less than two.

Due to the correlation between similarity and association, only low to moderate similarity words were chosen (Average Similarity Rating < 4). These pairs were matched across level of concreteness for average number of letters and written frequency. Pairs were first chosen for the dichotomous “associated/not associated” task. To create the blocks for this task, 30 concrete words pairs were quasi-randomly selected (20 associated and 10 unassociated), and 30 abstract pairs were similarly chosen (20 associated and 10 unassociated). Thirty pseudo-word pairs were then created for a rhyming task. These pseudowords were matched to the semantic stimuli for number of letters. Pseudowords were taken from a dataset of stimuli previously used to study non-word reading and modified for American phonetics (Besner et al., 1981). These words were all phonologically and orthographically plausible. Because the words from this study were all one syllable, they were modified to match the longer semantic stimuli.

### Task

**FIGURE 1:**
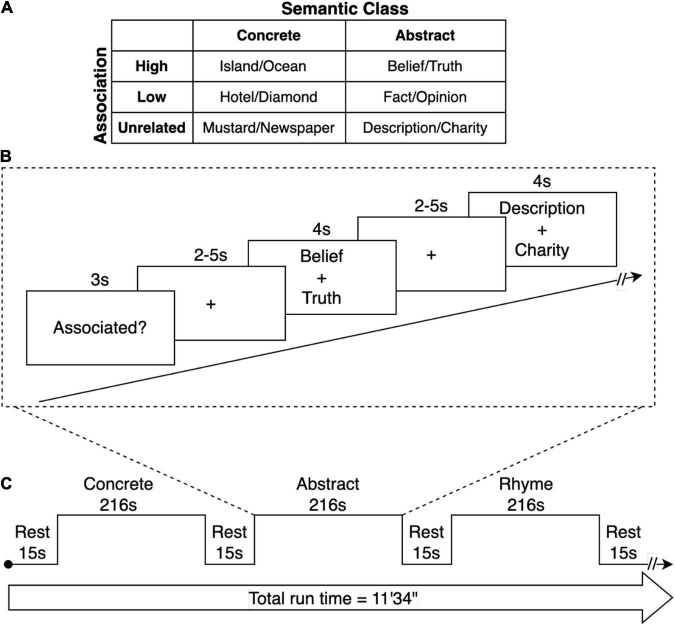
fMRI Paradigm. **(A)** Semantic stimuli consisted of pairs of words with high- or low-associative relationship or that were unrelated. Each semantic condition contained an equal number of abstract and concrete word-pairs. The phonemic control condition contained rhyming or non-rhyming word-pairs. **(B)** Trials consisted of a sequence of visual stimuli for each condition. At the onset of each condition, a word was presented for 3 s that with the task demand (Associated? or Rhyme?). Subsequently, word-pairs were presented for 4 seconds. This was followed by a period during which participants responded with a binary button press depending on whether the word-pair was judged to be related (associated) or unrelated. For the phonemic condition, participants determined whether the words rhymed. The period after each word-pair presentation was of random duration of 2 and 5 s (jittered), providing a variable lag between trials to enable subsequent event-related analysis. **(C)** A repeating block sequence was presented alternating between the concrete, abstract, and rhyme word-pair conditions, each 216 s in duration, with a 15 s rest period between each block.

Prior to scanning, participants completed an offline familiarization/practice task outside the scanner with word pairs that were not used in the trials. Participants were provided with clarification regarding any potential ambiguities in the directions. For the dichotomous “associated/not associated” task, participants were instructed to push a button indicating whether they believed the two words were associated. They were informed that “associated” referred to whether two words were commonly used together, and they were provided with examples of words that may be dissimilar but are commonly associated (e.g., tongs, barbecue). For the rhyming task, participants were instructed to push a button indicating whether they believed the two pseudowords rhyme (see Figure 1).

Participants viewed stimuli on a monitor situated behind the scanner via a mirror slotted over the head coil. All stimuli were presented in Arial 48 font in the center of the screen via Eprime 2.0 Professional software. We synchronized scanning with stimulus presentation by time locking the radiofrequency (RF) pulse to the offset of each trial using a TTL synchronization box (Nordic NeuroLab, Bergen, Norway). That is, the synchronization box transduced the analog TTL pulse (a 5v square wave) into a digital signal (the number 5) which was read by the presentation computer’s serial port. E-prime was programmed to terminate any trial upon receipt of a “5” signal, corresponding to a new RF pulse. This procedure ensured that no cumulative timing errors were introduced into the experiment due to stimulus buffering.

Participants completed one structural and five functional sessions over the course of one hour. The first three functional scans used identical stimuli and consisted of the dichotomous “associated/not associated” and the rhyme task. Participants were instructed to treat each functional session as if it were new and not to try to recall what they had answered on previous trials. These first three runs were constructed as mixed block/event-related designs. That is, each run was comprised of three long blocks: a concrete association block, an abstract association block, and a rhyming block. The presentation order of these blocks was randomized within the run, and the presentation order of the individual trials was randomized within each block. Before each block, participants were presented with a rest period of 15 s. At the beginning of each block, a word was presented for 3 s to indicate the semantic decision to be made for that block (e.g., “Associated?”). Participants were then presented with the “associated” or “rhyme” trials. Each word pair was presented for 4 s, during which time participants pressed a button to indicate their decision regarding association or rhyming. Stimulus presentation was followed by a jittered fixation cross (2–5 s). Each dichotomous decision run was 11′34′′ in duration. The other two runs consisted of additional semantic tasks that are not included in the current study.

### Image acquisition

Images were acquired on a Philip 3 Tesla Achieva (Amsterdam, Netherlands) with a SENSE 32-channel head coil. Foam padding minimized head motion. Functional images were obtained with a 1-shot gradient echo interleaved EPI sequence (TR = 2000 ms; TE = 30 ms; FOV = 224 mm; matrix size = 64 × 64; 3.5 mm x 3.5 mm in-plane resolution, flip angle = 80*^o^*). Thirty-six 3.5 mm thick axial slices with no gap covering the whole brain were acquired. A high-resolution T1-weighted, 3D anatomical scan (TR = 7.0 ms; TE = 3.7 ms; FOV = 240 mm; FA = 8*^o^*; matrix size = 240 × 240; 170 × 1.0 mm slices) was obtained prior to functional imaging.

### Analysis

#### Behavioral data

Behavioral data were examined for consistency with the expected response (i.e., “accuracy”). Runs where the correlation between the expected and given responses was below 0.5 were not analyzed. The effect of age on response time and accuracy were investigated using a set of repeated measures analyses of covariance (MANCOVA). Notably, these analyses were performed on a subset of 35 participants due to an equipment malfunction in the recording of responses for three participants.

##### Generalized psychophysical interaction analyses

Data were analyzed using statistical parametrical mapping (SPM 12) software^1^ and the CONN-fMRI functional connectivity toolbox v15h (Whitfield-Gabrieli and Nieto-Castanon, 2012). Within each subject, the functional images were slice-time corrected and realigned using 6-parameter rigid body transformation. The T1 anatomical scan was segmented and normalized into Montreal Neurological Institute (MNI) space. This transformation was then applied to the functional images. Movement outliers (*z*-score > 2.5 SD from the mean power or >1 mm of movement) were identified using the ARTifact detection Tools software package (ART). Runs with greater than 52 outlier TRs (15% of the run) were excluded from further analysis. Four participants were excluded from analysis due to excessive movement. Functional runs were then smoothed with an 8 mm full-width half-maximum Gaussian kernel. Next the functional runs were denoised, such that potentially confounding temporal covariates and physiological noise were removed from the time series using the anatomical CompCor approach. Notably, the main effect of each condition was entered into the model as a potential confound, such that areas that were co-activated during task would not be mistaken as functionally connected. Additionally, a high bandpass filter of 0.008 Hz was applied and linear detrending was conducted. Finally, the generalized psychophysiological interaction (gPPI) analysis was performed for whole-brain seed-to-voxel analyses. In these analyses, bivariate temporal regressions were calculated between each seed (described below) and all other voxels in the brain.

##### Statistical analyses

Three cortical areas considered to either be part of the semantic network or that have been implicated in semantic processing served as seeds. These included the Anatomical Automatic Labeling (AAL) atlas regions of the left anterior middle temporal gyrus (aMTG), left angular gyrus (AG) and a combined region of left pars opercularis and left part triangularis (hereafter referred to as the left inferior frontal gyrus (IFG). A ventral anterior temporal lobe seed was considered, but examination of the EPI data revealed some artifact in this area. Seed-to-voxel statistical connectivity maps for each seed were created for the following contrasts: (1) abstract + concrete > rhyme, (2) abstract > rhyme, (3) concrete > rhyme. Whole-brain results were considered significant at the voxel-level threshold of *p* < 0.001, and a cluster level threshold of *p* < 0.05, FWE. The extent and distribution of connectivity of aMTG, AG, and IFG for the abstract and concrete was examined next. The total number of voxels in clusters in the frontal, parietal, temporal, and occipital lobes of each hemisphere that were significantly connected (FDR corrected) with each of the seeds was derived by summing the voxels per cluster for the abstract and concrete word conditions. The number of significantly connected voxels for each lobe of the right and left hemispheres was contrasted (FWE corrected) between the abstract and concrete semantic conditions. Effects of age on functional connectivity differences between conditions was then examined with mean-centered age entered into the gPPI model as a second level covariate.

## Results

### Connectivity of the semantic system

#### Semantic versus rhyme processing

**FIGURE 2:**
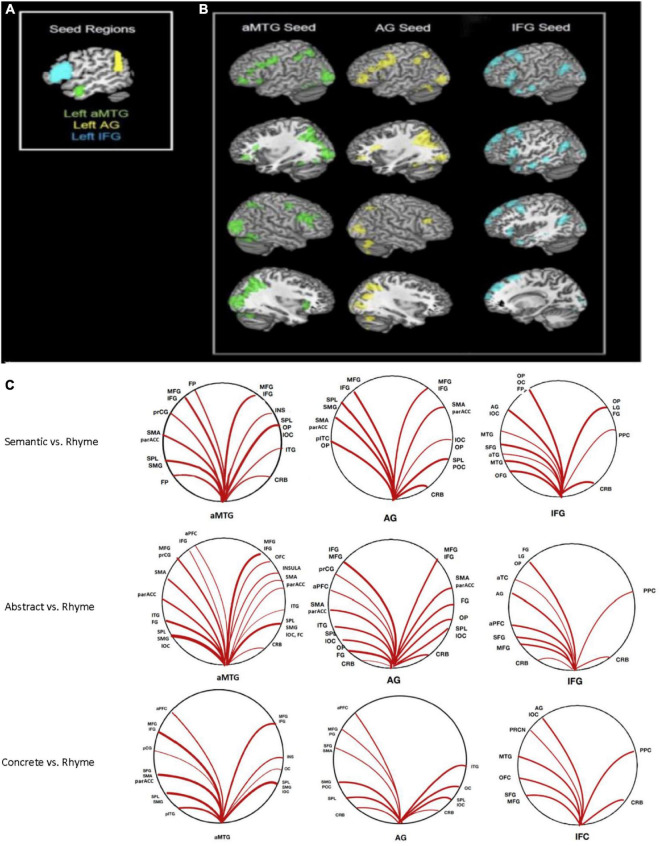
Seed-to-voxel connectivity: **(A)** Seeds in anterior middle temporal gyrus (aMTG), angular gyrus (AG), and inferior frontal gyrus (IFG). **(B)** Regions with significant connectivity with each seed during semantic processing (abstract + concrete combined). **(C)** Graphic representation of significant connections (*p* < 0.05, FWE corrected) to each seed for contrasts of overall, abstract, and concrete semantic relative to the rhyme condition.

Significant connectivity was evident among cortical areas, though this varied by cortical seed with connectivity greater during semantic processing for some areas. The most relevant findings are discussed below with a full description of all significant connections provided in Tables 1–3. Figure 2 shows ROIs with functional connectivity that reached FDR-corrected statistical significance (*p* < 0.05) from each of the three seed regions (aMTG, AG, IFG) for contrasts of overall semantic, concrete, and abstract word processing relative to the phonemic rhyme processing.

**TABLE 1:** Whole brain analyses of seed-to-voxel analyses for semantic processing relative to rhyme, seeding the l aMTG, l AG, and l IFG.

Seed	Brain region	Cluster size	Peak-T	Peak MNI		
				*X*	*Y*	*Z*
aMTG	**Left Hemisphere Connectivity**					
	SPL, SMG, lOC	891	5.80	–26	–60	62
	OP	576	7.27	–30	–94	2
	SMA, ParCC	442	5.58	–6	14	48
	MFG, IFG	327	5.85	–36	32	18
	PrCG	295	5.74	–40	–2	38
	FP **Right Hemisphere Connectivity**	195	5.23	–40	52	–4
	SPL, OP, lOC	1974	8.72	24	–92	4
	MFG, IFG	498	7.09	52	24	34
	CRB	164	5.92	8	–84	–24
	ITG	127	4.93	50	–56	–16
	INS, IFG	122	5.17	34	26	–2
L AG	**Left Hemisphere Connectivity**					
	MFG, IFG, prCG	1818	5.79	–40	48	–2
	pITC, OP	1242	7.06	–28	–88	–2
	SPL, SMG	1215	5.57	–28	–50	38
	**Bilateral Connectivity**					
	SMA, ParCC	186	5.91	–4	6	82
	**Right Hemisphere Connectivity**					
	CRB	1011	6.47	40	–60	–26
	OP, lOC	846	6.81	24	–84	2
	SPL, OC	560	5.09	28	–52	48
	lOC	213	5.11	32	–66	26
L IFG	**Left Hemisphere Connectivity**					
	SFG	680	7.58	–10	50	36
	AG, lOC	617	6.16	–48	–68	26
	IFG, OFG	346	7.10	–48	18	14
	MFG	257	6.37	–36	6	48
	MTG	158	4.75	–54	–12	–18
	aTG	88	4.83	–46	12	–30
	**Bilateral Connectivity**					
	OP, LG, FG	721	7.71	–12	–86	–14
	**Right Hemisphere Connectivity**					
	CRB	1207	5.71	36	–76	–36
	pPC	209	5.37	28	–42	20

Clusters significant at *p* < 0.05, FWE-corrected. See Table 5 for key showing abbreviations for each ROI.

**TABLE 2:** Seed-to-voxel analyses for abstract word relative to rhyme processing.

Seed	Brain region	Cluster size	Peak-T	Peak MNI		
				*X*	*Y*	*Z*
aMTG	**Left Hemisphere Connectivity**					
	SPL, SMG, lOC	1683	8.16	–26	–88	6
	MFG, prCG	497	6.29	–40	–2	38
	pITC, FG	292	5.77	–52	–56	–16
	IFG	265	5.55	–38	28	18
	aPFC	231	5.38	–44	52	–4
	**Bilateral Connectivity**					
	SMA	211	5.00	–6	0	64
	ParCC	204	5.46	6	20	40
	**Right Hemisphere Connectivity**					
	SPL, SMG, lOC, OP, FG	2835	8.73	28	–88	12
	MFG, IFG	404	6.10	56	22	36
	lOC, pITC	272	5.68	44	–62	–12
	pITC	314	5.32			
	OFG, INS	145	4.58	36	24	–4
	CRB	113	4.19	24	–70	–50
	prCG	95	4.67	46	–6	62
AG	**Left Hemisphere Connectivity** MFG, PrCG					
		1944	6.19	–42	28	22
	SPL, lOC	1707	6.37	–24	–62	28
	OP, FG	674	6.12	–18	–94	–6
	pITC	314	5.32	–48	–58	–14
	IFG **Bilateral Connectivity**	255	7.09	–50	20	12
	SMA, ParCC	384	5.40	–6	6	62
	**Right Hemisphere Connectivity**					
	CRB	659	5.81	12	–80	–22
	OP, FG	618	6.28	20	–90	2
	SPL, lOC	437	4.90	24	–50	50
	IFG, MFG	187	4.87	50	30	28
IFG	**Left Hemisphere Connectivity**					
	OP, LG, FG	830	6.99	–10	–92	04
	aPFC	402	6.58	–12	58	34
	AG, lOC	328	5.55	–40	–68	28
	MFG	353	7.30	–40	10	48
	SFG pMTG, STG	240 212	5.64 5.49	–12 –56	26 –32	66 6
	aTG	79	4.85	–48	10	–30
	**Right Hemisphere Connectivity**					
	CRB	582	5.30	36	–76	–36
	pPC	106	4.47	26	–36	22

Clusters shown are significant at *p* < 0.05, FWE-corrected. inferior temporal gyrus (IFG); angular gyrus (AG); anterior medial temporal cortex (aMTG). See Table 5 for key showing abbreviations for each ROI.

**TABLE 3:** Seed-to-voxel analyses for concrete word relative to rhyme processing.

Seed	Brain region	Cluster size	Peak-T	Peak MNI		
				*X*	*Y*	*Z*
aMTG	**Left Hemisphere Connectivity** SPL, SMG, lOC MFG, IFG	697 671	5.01 5.25	–30 –36	–60 32	62 18
	SFG, SMA, ParCC	439	5.55	–8	18	46
	OC	178	5.02	–36	–98	0
	aPFC	139	4.34	–42	46	–6
	pITC	136	5.61	–50	–44	–20
	PrCG	74	4.28	–40	–2	38
	**Right Hemisphere Connectivity**					
	SPL, SMA, lOC	679	6.67	28	–38	48
	MFG, IFG	451	6.91	52	26	32
	OC	238	5.75	34	–84	16
	INS	182	5.10	38	26	10
AG	**Left Hemisphere Connectivity** OC, CRB	750	7.13	–36	–76	–24
	SMG, OC	415	4.97	–30	–62	62
	MFG, PrCG	304	4.42	–42	4	38
	pITC SFG, SMA aPFC	560	6.37	40	–60	–26
		124	6.47	–6	9	60
		87	4.58	–40	52	–14
	**Bilateral Connectivity**					
	CRB	149	4.69	12	–80	–22
	**Right Hemisphere Connectivity**					
	OC	534	5.84	32	–92	6
	SPL, lOC	374	4.94	34	–54	46
IFG	**Left Hemisphere Connectivity**					
	SFG, MFG	1652	7.82	–10	54	34
	AG, lOC	733	6.48	–44	–66	14
	PRCN	202	5.04	–6	–56	14
	MTG	153	4.77	–56	–14	–16
	OFG	123	5.54	–34	30	–12
	IFG	93	5.59	–52	24	8
	**Right Hemisphere Connectivity**					
	CRB, LG	1624	6.77	42	–72	–36
	pPC	105	5.03	28	–34	24

Clusters shown are significant at *p* < 0.05, FWE-corrected. IFG, inferior temporal gyrus; AG, angular gyrus; aMTG, anterior medial temporal cortex. See Table 5 for key showing abbreviations for each ROI.

**TABLE 4:** Cluster sizes of connected areas by semantic condition.

	Left**		Right	
	Abstract	Concrete	Abstract	Concrete
**aMTG**				
Frontal	1165.3 (406.5)	1235.7 (426.0)	644.4 (134.0) **	451.1 (165.3)
Temporal	265.4 (222.3)	136.0 (235.3)	314.3 (240.5)	182.2 (226.1)
Parietal	1483.4 (452.4)**	697 (282.8)	2535.6 (520.1) **	679.0 (390.7)
Occipital	245.2 (175.5)*	178.2 (134.5)	435.3 (198.2)*	274.6 (189.0)
ACC	145.8 (75.5)*	95.6 (43.4)	55.6 (43.4)	–
**AG**				
Frontal	2214.2 (555.2)**	614.2 (435.0)	337.2 (342.0)	486.9 (335.3)
Temporal	314.4 (185.5)	560.1 (228.3)	–	–
Parietal	1655.3 (389.4) **	305.4 (195.6)	405.4 (265.0)	375.3 (285.0)
Occipital	735.7 (195.6)*	602.2 (210.0)	755.4 (188.9)*	622.0 (166.2)
ACC	124.2 (89.3)	–	95.0 (77.6)	–
CRB	^–^	250.4 (165.4)	659.4 (275.0)**	120.2 (92.3)
**IFG**				
Frontal	1210.0 (678.3)	2024.7 (955.4) **	–	^–^
Temporal	291.4 (106.3)	276.5 (100.2)	–	–
Parietal	239.6 (189.3)	840.6 (335.4) **	105.7 (97.0)	106.8 (89.5)
Occipital	1100.0 (458.5)	933.4 (386.4)	–	–
CRB	–	–	582.3 (245.0)	844.6 (306.1)**

The total number of voxels in cortical clusters significantly connected with the left aMTG, AG, and IFG seeds were aggregated and summed by lobe (frontal, temporal, parietal, and occipital) by hemisphere. Mean number of voxels are presented with standard deviation in parentheses. Significant differences in aggregate lobar volumes between the abstract and concrete conditions are indicated: **p < 0.01, *p < 005. Lobes in which no clusters were significantly connected to a seed are indicated by – . Greater connectivity was evident during abstract semantic processing of the AG to frontal, parietal, and occipital areas, and of the aMTG to parietal and occipital areas. Conversely, the left IFG was more strongly connected with the left parietal and occipital areas and right frontal and cerebellar areas during concrete semantic processing. Connectivity of the IFG with the anterior cingulate cortex (ACC) and cerebellum (CRB) differed in a similar manner abstract and concrete semantic processing.

**TABLE 5:** Abbreviations of ROIs.

ACC	Anterior cingulate cortex
AG	Angular gyrus
aMTG	Anterior middle temporal gyrus
aPFC	Anterior prefrontal cortex
aTC	Anterior temporal cortex
aTG	Anterior temporal gyrus
CRB	Cerebellum
CN	Caudate nucleus
CUN	Cuneus
FC	Fusiform cortex
FP	Frontal pole
FG	Fusiform gyrus
HIP	Hippocampus
IFG	Inferior frontal gyrus
IPL	Inferior parietal lobule
INS	Insula
ITG	Inferior temporal gyrus
lOC	Lateral occipital cortex
LG	Lingual gyrus
MFG	Middle frontal gyrus
MPC	Middle parietal cortex
MTG	Middle temporal gyrus
OC	Occipital cortex
OP	Occipital pole
OFC	Orbital frontal cortex
OFG	Orbital frontal gyrus
ParCC	Paracingulate cortex
PCC	Posterior cingulate cortex
pCG	Precentral gyrus
pITC	Posterior inferior temporal cortex
pITG	Posterior inferior temporal gyrus
POC	Posterior occipital cortex
pPC	Posterior parietal cortex
PRCN	Precuneus
SFG	Superior frontal gyrus
SMG	Supramarginal gyrus
SMA	Supplementary motor area
SPC	Superior parietal cortex
SPL	Superior parietal lobule
STL	Superior temporal lobule
THAL	Thalamus
TTG	Transverse temporal gyrus

When cortex rather than gyrus is indicated, the cluster extended beyond the boundaries of the gyrus.

#### Anterior middle temporal gyrus seed

Greater connectivity during semantic compared to phonemic rhyme processing between the left aMTG and frontal cortical and posterior heteromodal association areas was evident. This included connectivity with areas responsible for visual processing (i.e., ventral/dorsal visual pathways) and motor processing (i.e., left supplementary motor area, left precentral gyrus, left frontal pole, and bilateral frontal cortices).

#### Angular gyrus seed

Greater connectivity during semantic processing compared to phonemic rhyme processing was evident in frontal and posterior language regions (i.e., left inferior frontal gyrus, left supramarginal gyrus, and left superior parietal lobule), as well as secondary association areas.

#### Inferior frontal gyrus seed

The left IFG seed, which incorporated pars triangularis and pars opercularis, showed greater connectivity with frontal and temporal language regions during semantic processing as compared to phonemic rhyme processing. As hypothesized, another cluster extended from the left AG to the left lateral occipital cortex. Clusters of increased connectivity were also located in the primary and secondary visual association cortex.

#### Abstract versus rhyme word processing

Significant connectivity was evident between seeds and cortical areas, though the specific pattern varied by cortical seed. There was greater connectivity during the semantic processing of abstract words compared to phonemic rhyme processing across multiple brain regions relative to the aMTG, AG, and IFG. Table 2 lists ROIs with functional connectivity from these three seeds that reached FWE-corrected statistical significance (*p* < 0.05).

#### Anterior middle temporal gyrus seed

Connectivity from the left aMTG from the contrast of response to abstract versus rhyming words indicated similar functional connectivity as in the overall semantic – rhyme map. Increased connectivity to both frontal language regions and heteromodal association cortices was observed. When examining the cluster size for all connected regions (left hemisphere, right hemisphere, and bilateral clusters), the extent of connectivity for abstract semantic vs. rhyme processing was increased compared to the overall semantic-rhyme contrast (Table 2).

#### Angular gyrus seed

Connectivity with the left angular gyrus was similar to the overall semantic to rhyme contrast, but with slightly decreased extent of the connectivity to parieto-occipital regions and increased extent of connectivity to frontal regions. As hypothesized, there was increased connectivity with frontal regions during abstract word processing.

#### Inferior frontal gyrus seed

Similar cortical areas were functionally connected to the IFG seed for the abstract vs. rhyme and semantic vs. rhyme contrasts. Significant connectivity with frontal gyri was observed, with reduced posterior connectivity as compared to overall semantic vs. rhyme contrasts.

#### Concrete versus phonemic processing

Greater connectivity of the aMTG, AG, and IFG and multiple cortical areas was evident during concrete as compared to phonemic rhyme word processing. Table 3 provides the coordinates and the number of voxels in clusters containing the ROI for areas with significant connectivity the three seeds that reached FWE-corrected statistical significance (*p* < 0.05).

#### Anterior middle temporal gyrus seed

Connectivity of left aMTG seed revealed a connectivity map that was similar though less extensive to the corresponding abstract connectivity map. The largest clusters were located in the left and right posterior perisylvian cortex, with additional clusters in areas responsible for visual processing (i.e., occipital lobe) and motor processing (i.e., supplementary motor area).

#### Angular gyrus seed

Connectivity of left AG to other brain regions was similar, though less extensive than the corresponding abstract analysis. The extent of connectivity was decreased in both frontal and posterior cortex. As predicted, there was bilateral connectivity of local cortex, including left and right lateral occipital cortex and superior parietal lobule.

#### Inferior frontal gyrus seed

Compared to the abstract maps, there was decreased extent of local connectivity (i.e., connectivity between adjacent regions) in the left IFG, decreased anterior temporal lobe connectivity, and slightly increased extent of posterior perisylvian connectivity during concrete word processing vs. rhyme processing.

#### Abstract versus concrete word processing

Comparison of connectivity strengths of the aMTG, AG, and IFG with cortical ROIs between the abstract and concrete processing conditions revealed a limited number of significant differences in connectivity following FDR correction, though a few differences were found. The left AG had greater connectivity with areas of the frontal cortex, including the inferior frontal gyrus of the left hemisphere and the inferior frontal gyrus, middle frontal gyrus, supplementary motor area, and paracingulate cortex of the right hemisphere during abstract semantic processing (*p* < 0.05, FDR corrected). The aMTG had greater connectivity with the orbital frontal cortex, supplementary motor area, insula, and paracingulate of the right hemisphere also during abstract processing (*p* < 0.05, FDR corrected). Conversely, during concrete processing, the IFG had greater connectivity with the left lingual and fusiform gyri, and the occipital pole (*p* < 0.05, FDR corrected).

Abstract and concrete processing elicited significantly different distributions of connectivity from aMTG, AG, and IFG to cortical clusters of the left vs. right hemispheres. The number of voxels in cortical clusters significantly connected with each of the three seeds was much greater in the left hemisphere than the right hemisphere (*p* < 0.001). Table 4 summarizes differences between the abstract and concrete conditions with respect to the number of voxels in clusters that were significantly connected with the aMTG, AG, and IFG in the left and right hemisphere by lobe of the cortex.

The extent of significant connectivity of the left aMTG, AG, and IFG with cortical clusters in the left and right frontal, parietal, temporal, and occipital lobes (i.e., the total number of voxels in connected cluster per lobe) differed between the abstract and concrete semantic conditions (*p* < 0.05, FWE corrected). Bilateral connectivity from left AG to frontal, parietal, occipital, and anterior cingulate clusters was greater during abstract semantic processing. During abstract semantic processing, greater connectivity was also evident between the left aMTG and parietal and occipital clusters and bilaterally with occipital and anterior cingulate clusters. In contrast, the IFG had more extensive connectivity with cortical areas of the left hemisphere during concrete as opposed to abstract semantic processing, whereas IFG connectivity to areas of the right hemisphere did not differ significantly between the semantic conditions. There was greater connectivity between the AG and cerebellum during abstract semantic processing. However, there was greater connectivity between the IFG and cerebellum during concrete processing. Differences between condition were not found for connectivity between the aMTG and cerebellum.

##### Age effects on semantic performance

The effect of age on response time were compared across the study conditions (i.e., abstract semantic, concrete semantics, and phonemic). The overall model approached significance, Wilk’s Lambda = 0.83, *F*(2, 32) = 3.19, *p* = 0.06. Mauchly’s test demonstrated that the assumption of sphericity had been met (*p* = 0.152). There was a significant main effect of pair type, *F*(2,66) = 4.031, *p* < 0.05, and of age on response time, *F*(1,33) = 7.49, *p* < 0.004. There was additionally a significant interaction of pair type by age, *F*(2,66) = 3.38, *p* < 0.046. ***Post hoc*** pairwise comparisons revealed that concrete word processing was significantly faster than either abstract word processing (*p* < 0.001) or phonemic processing of rhyming words (*p* < 0.001), but abstract and phonemic processing times were not significantly different. Univariate parameter estimates revealed that age significantly predicted response time for both semantic word pairs (abstract and concrete) but not for rhyming: Abstract: β = 13.24, *p* < 0.01; Concrete: β = 8.38, *p* < 0.01; Rhyme: β = 5.17, *p* = 0.19. Greater age was associated with greater response times during abstract semantic processing.

Analyses of the effect of age by experimental condition on accuracy of semantic judgments did not reveal significant main effects for age [*F*(1,33) = 1.46, *p* = 0.24]. All participants showed a relatively high level of accuracy (Abstract: 79.3 ± 8.4%, Concrete: 82.4 ± 7.2%, Phonemic: mean = 93.5 ± 5.2%). There was a significant difference in accuracy between conditions [*F*(2,32) = 3.30, *p* = 0.049]. *Post hoc* comparisons indicated greater accuracy on the phonemic condition compared to the abstract [*t*(1) = 8.20, *p* < 0.001] and concrete [*t*(1) = 8.20, *p* < 0.001] conditions. Accuracy on the abstract and concrete conditions did not differ significantly [*t*(1) = 1.6, *p* = 0.12]. Stronger accuracy on the phonemic condition was expected, as word-pairs either rhymed or they did not, whereas semantic judgments about word associations is more subject to individual differences in perceived relatedness of the words.

##### Age and functional connectivity

**FIGURE 3:**
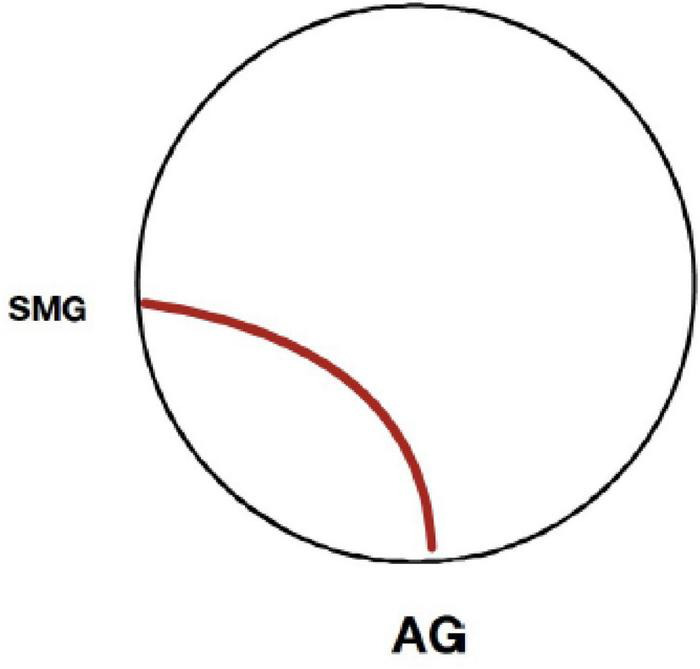
Seed-to-voxel connectivity as a function of age. For the overall semantic and concrete semantic conditions relative to the phonemic (rhyming) condition, only one connection was statistically significant as a function of greater age (*p* < 0.05, FWE corrected). This functional connection was between adjacent cortical areas of the left angular gyrus and supramarginal gyrus. No significant connections were found for the abstract semantic condition as a function of greater age.

Greater age was associated with increased connectivity between the left AG and left SMG during overall semantic processing (*t* = 6.18, *p* < 0.01, FWE corrected) and concrete word processing (*t* = 6.30, *p* < 0.01, FWE corrected) compared to phonemic processing (see Figure 3).

## Discussion

The current study examined functional connectivity between neural systems during the semantic processing of concrete and abstract words within a sample of middle aged to older adults. We will first describe the general connectivity findings within the context of the general semantic system, looking widely at semantic vs. rhyme processing and then at abstract and concrete processing more narrowly. We will then examine the age-related findings, including implications of possible stability of semantic systems in aging.

### Functional connectivity during semantic processing

With regards to semantic processing in general, we found that functional connectivity between cortical areas previously implicated as semantic processing hubs (angular gyrus and anterior temporal lobe, including the temporal pole) and other cortical association areas, varied as a function of task demand. Consistent with the study hypotheses, the left aMTG and left AG had increased functional connectivity with multiple cortical areas during semantic processing tasks, but these two hubs were not significantly connected with each other. For both seeds, we see two convergent networks appear: the first is composed of areas traditionally included in the semantic network and the second is a distributed network of areas typically associated with attention and working memory, both specific to and beyond the context of semantic processing.

First, both the left aMTG and left AG were significantly connected to areas thought to underscore more traditional language processes, including occipital areas of the ventral visual stream and the left inferior temporal gyrus. The former has been implicated in the processing of visual aspects of words, specifically the role of visual imagery in building word meaning (Binder et al., 2009; Meyer and Damasio, 2009). The latter has traditionally been linked to word reading but more recently has been implicated in semantic processing specifically (Badre et al., 2005). More notable was the prominent frontal and parietal connectivity for both seeds. The seeded regions were functionally connected to large areas of the left dorsal medial prefrontal cortex, supplementary motor area, and IFG. These connected regions engage in goal-directed working memory more broadly, with the dorsal medial prefrontal cortex and supplementary motor area implicated in goal-directed semantic activity specifically (Badre et al., 2005; Ye and Zhou, 2008, 2009; Binder et al., 2009; Jefferies, 2013; Noonan et al., 2013). The role of the IFG in semantic functioning is broadly thought of as contributing to semantic control, with the different subregions contributing to different components of control. The left AG was connected only to pars triangularis, while the aMTG extended more anteriorly to include both pars triangularis and pars opercularis. The implications of this distinction are subtle, but they suggest that both the left AG and aMTG are working in concert with selection demands, but the aMTG is additionally connected to regions sensitive to selection amongst associated competitors. Thus, both the left AG and left aMTG demonstrated connectivity with bilateral frontal-executive control networks (with the left AG demonstrating a tendency toward left lateralization). They additionally were both connected to large areas of the posterior parieto-occipital cortex, with clusters spanning from the superior parietal lobules to the supramarginal gyri and lateral occipital cortex. As with the connected frontal regions, these regions have been thought of as both general working memory engines, though they more specifically relate to manipulation of information (Koenigs et al., 2009). Functional connectivity with frontal and parietal cortical areas suggests involvement of the frontal-parietal control network (Badre et al., 2005; Davey et al., 2016).

While the left aMTG and left AG showed increased connectivity during semantic processing with many of the cortical areas, there were also some differences in their connectivity. The left aMTG had more bilateral connectivity, with relatively increased right hemisphere frontal and parietal connectivity. The left AG, on the other hand, had more local connectivity with the supramarginal gyrus and inferior parietal lobule. This increased local connectivity is consistent with previous reports of higher local clustering of the angular gyri in older adults (Sala-Llonch et al., 2014). Additionally, these differences in functional connectivity correspond with underlying structural connectivity of both regions. The white matter tracts of the uncinate fasciculus and inferior frontal-occipital fasciculus connect the anterior temporal lobe and frontal regions, while the superior longitudinal fasciculus connects the dorsal medial prefrontal cortex and superior frontal gyrus to the angular gyrus (Badre et al., 2005; Catani and Mesulam, 2008). The left angular gyrus is structurally connected to secondary association cortical areas (Badre et al., 2005). That significant functional connectivity was evident between the left AG and secondary association areas of the visual cortex may reflect the nature of the stimuli, which were chosen to (1) match across psycholinguistic variables, and (2) maximize differences between concrete and abstract concepts. Concrete concepts are more strongly linked to the physical attributes than are abstract concepts, disproportionately taxing the visual systems, resulting in greater functional connectivity with secondary association areas of the visual cortex. Conversely, frontal cortical areas are typically more responsive to the control of semantic processing (Badre et al., 2005).

The connectivity of the left IFG corresponded most closely to expected regions of semantic processing. This region was connected both to the left AG and the left anterior temporal lobe, the two hypothesized hub regions of the semantic network. It additionally was connected to several areas along the left MTG, an area that has been implicated in heteromodal association and integration of semantic concepts (Binder et al., 2009) as well as the ventral medial prefrontal cortex, which may mediate the emotional components of language (Badre et al., 2005; Binder et al., 2009). The left IFG was functionally coupled to adjacent inferior frontal and orbitofrontal cortices, both of which are associated with a wide-array of higher order semantic control tasks, as well as the dorsomedial prefrontal cortex, which is specifically associated with goal-directed semantic control tasks (Thompson-Schill et al., 1997; Noonan et al., 2013). Thus, the left IFG was connected to traditional higher order semantic control networks, but also to regions important for lower order semantic processing and integration. These results correspond to previous reports of functional connectivity between the left IFG and a distributed semantic control network in a younger cohort (Davey et al., 2016). The current findings also highlight the importance of semantic control in response to this relatively easy task.

To summarize, the left aMTG and left AG had similar connectivity maps. In both, the semantic task elicited strong connectivity between hypothesized hub regions and a frontal-parietal control network. These results support previously reported task-related connectivity between the semantic hubs and areas associated with goal-oriented activity. The left IFG, meanwhile, was more broadly connected to regions of the semantic network, semantic control network, and multidomain control network during a semantic task.

### Functional connectivity during abstract and concrete processing

Regardless of demand for abstract or concrete semantic processing, three seeds in the left cortex (aMTG, AG, and IFG) were functionally connected to many of the same areas distributed bilaterally across the cortex. The aMTG and AG exhibited significant functional connectivity with multiple areas in frontal, parietal, temporal, and occipital cortices during both abstract and concrete semantic processing. The IFG had significant connectivity with other areas of the frontal cortex, and also with posterior cortical areas of the parietal and occipital cortex during both concrete and abstract semantic processing. Each of the seeds also had significant connectivity with each of the hypothesized hubs during both semantic conditions. Accordingly, overall functional connectivity of the aMTG, AG, and IFG with cortical areas was similar during both abstract and concrete semantic processing relative to phonemic processing. This finding provides supporting evidence that semantic hubs (aMTG and AG) and an area for semantic control (IFG) in the left cerebral cortex act in conjunction with broad cortical association systems for semantic processing of both concrete and abstract words.

Yet, differences between the abstract and concrete semantic conditions were also evident with respect to the strength of connectivity of the left aMTG, AG, and IFG and cortical ROIs, and the extent of connectivity between these seeds and cortical clusters. Differences in the extent of connectivity was most obvious, as visual inspection of the connectivity maps from aMTG, AG, and IFG suggested more extensive overall connectivity with cortical areas during the processing of concrete words. Furthermore, differences in the cortical distribution of connectivity also seemed to exist. These observations were supported by statistical comparisons of the concrete and semantic processing conditions as both the strength and extent of connectivity differed as a function of semantic condition for each of the seeds. Larger differences in the extent of connectivity were evident between the semantic conditions compared to the strength of connectivity between the seeds and specific cortical coordinates.

When the abstract and concrete words were collapsed into a single semantic condition, much more extensive connectivity of the left aMTG, AG, and IFG with cortical and subcortical areas of the ipsilateral left hemisphere was observed compared to areas of the right hemisphere. Yet, comparison of the abstract and concrete semantic conditions revealed greater complexity with respect to the extent of functional connectivity cortical areas with aMTG, AG, and IFG. Significant differences were evident in the extent of connectivity of aMTG, AG, and IFG with left versus right frontal, temporal, parietal, and occipital areas as a function of semantic condition. The most obvious difference was that cortical areas of the right hemisphere had more extensive connectivity to the putative semantic hubs (aMTG, AG) during abstract semantic processing (>2.5x). This was not the case for connectivity from the IFG as the extent of connectivity with right cortical areas were not significantly different between semantic conditions, and the extent of connectivity between IFG and the left cerebellum was greater during concrete compared to abstract semantic processing. These findings suggest lateral asymmetry in the extent and distribution of connectivity for aMTG and AG as compared to IFG. These findings may seem contradictory to the more extensive connectivity with left cortical areas during semantic processing overall. However, these differences in connectivity with right and left cortical areas likely reflects connectivity during semantic processing when compared to phonemic processing versus connectivity as a function of word concreteness-abstractness. That the extent of right cortical connectivity with aMTG, AG and IFG differed is likely a manifestation of the roles played by each of these areas for semantic processing.

As a putative semantic control area, the IFG facilitates higher-order semantic processing for the sequencing, organizing, integrating, or synthesizing of concepts. The fact that IFG connectivity differences between semantic conditions were evident is noteworthy given that making semantic judgments about word associations or relatedness is not very demanding from the standpoint of semantic complexity. The IFG seems to be engaged and differentially connected to multiple cortical areas even when there is minimal demand for higher-order semantic processing. The left IFG was most strongly connected with ipsilateral cortical areas during concrete semantic processing, a result that was unexpected and somewhat counterintuitive, as this task theoretically has less demand for executive processes than abstract word processing. This finding may be a manifestation of the left hemisphere’s specialization for language and semantic functions. When there is minimal demand for higher-order semantic processing of concrete concepts, the IFG’s strongest connectivity with left posterior cortical areas likely reflects the relatively automatic engagement of sensory association areas. In contrast, during the more demanding cognitive processing automaticity is less possible (Kubler et al., 2006), such as with abstract concepts derived from associations that are not strongly not strongly linked to the specific physical attributes of experience. The more extensive engagement of cortical areas of the frontal cortex and the right hemisphere cortical with aMTG and AG during abstract word processing is logical given the greater physically ambiguous and conceptual complexity of abstract associative information.

The extent of intra-hemispheric connectivity of the left aMTG, AG, and IFG with left cortical areas also differed during abstract and concrete semantic processing and likely is influenced by similar factors. The extent of connectivity of the aMTG with cortical clusters of the parietal and occipital lobes was much greater during abstract semantic processing. Connectivity of the left AG to both frontal and parietal-occipital areas was more extensive during abstract semantic processing. The extent connectivity of the aMTG with parietal-occipital was also much greater during abstract semantic processing, but this was not the case for connectivity with frontal areas. This finding was unexpected given that the anterior temporal cortex plays an important role in the processing of abstract concepts (Hoffman et al., 2015), and the frontal anterior cingulate cortices important for higher cognitive functions, such as abstract reasoning (Reverberi et al., 2005; Jung et al., 2022), problem solving (Grossman et al., 2002; Newman et al., 2003; Cazalis et al., 2006; Feng et al., 2021), judgment and decision making (Zysset et al., 2002; Cunningham et al., 2004; Beer et al., 2010; Zander et al., 2016), creativity (Gonen-Yaacovi et al., 2013), the supervisory control of attention and intention (Shallice and Burgess, 1996; Cohen et al., 1999), planning (Goel and Grafman, 1995; Gonen-Yaacovi et al., 2013), goal-directed and generative action and cognition (Cohen et al., 1999; Baldo et al., 2001; Reverberi et al., 2005). Accordingly, it would be reasonable to infer that the aMTG would be more extensively connected to frontal cortex when processing abstract words. That this was not the case may again reflect the limited demand for semantic control when making semantic associative judgments. The current finding largely corresponds with previous fMRI findings regarding connectivity of a ventral anterior temporal seed with frontal areas during a similar association task involving concrete semantic judgments (Jackson et al., 2016).

While significant connectivity was also evident between the three seed areas and multiple cortical regions during concrete semantic processing, it was less extensive. This finding suggests that semantic judgments about abstract word associations can be accomplished via semantic control of the frontal cortex communicating with the AG. Interaction between aMTG and IFG may be less prominent for this type of semantic task. The significant connectivity of the left angular gyrus with multiple cortical areas suggests that it plays an important role in semantic processing of both abstract and concrete concepts, a finding consistent with models that posit two semantic hubs: the aMTG and AG (Reilly et al., 2016). The differential connectivity of the AG additionally corresponds to work that has extended the semantic control network to include more posterior perisylvian regions. Indeed, the dorsal angular gyrus specifically is routinely recruited for semantically challenging tasks (Noonan et al., 2013). However, the exact nature of its contribution to complex processing is still unclear. The role of the angular gyrus in thematic association processing may additionally explain its preferential role in abstract processing, as association has been hypothesized to underscore acquisition of abstract concepts (Badre et al., 2005; Price et al., 2015).

The left IFG similarly demonstrated different connectivity maps for concrete and abstract words. Both were significantly connected to the left angular gyrus (though slightly more extensive for concrete words) and areas of the left temporal lobe. Contrary to our hypotheses, however, concrete word processing elicited more connectivity between the left IFG and other areas of the frontal cortex, including the left superior frontal gyrus and dorsomedial prefrontal cortex. Thus, concrete words elicited more connectivity between the left IFG, and regions implicated in semantic control. Abstract words, on the other hand, elicited stronger connectivity between the IFG and the lateral temporal cortex, including the posterior and anterior middle temporal gyrus. To the extent that the left anterior temporal lobe was a hypothesized hub of higher order abstraction, this result was in line with expected outcomes. However, the connectivity between the left IFG and the temporal lobe was more extensive than predicted.

In sum, the current findings support the conclusion that more than one cortical area play key roles in semantic processing, and that these areas have extensive functional connectivity with secondary cortical association during semantic task performance. That greater connectivity from the left AG, aMTG, and IFG to secondary cortical association areas occurred during semantic processing is noteworthy given the limited demand for semantic control, generative semantic production, or higher-order operations involving semantic integration or problem solving. The task simply required judgments to be made about the relatedness of word pairs with responses consisting of a binary button press of yes or no. Yet, even when making this type of simple semantic judgment, significantly greater functional connectivity from AG, aMTG, and IFG compared to when the task involved phonological processing and judgments about words rhyming. The finding with respect to connectivity from the left IFG suggests that functional connectivity with this frontal cortical area is engaged even when tasks do not require intensive semantic control. Given that the extent and strength of connectivity of primary cortical semantic hubs with secondary cortical association areas depends on the demands of the task to be performed (Binder et al., 2009; Bonner et al., 2013; Reilly et al., 2016), it seems likely that tasks that require greater semantic control (e.g., generative semantic production) would elicit even stronger connectivity with areas in the frontal cortex, in particular the IFG. This conceptualization regarding the role of the IFG is with models of semantic processing that posit multiple demand systems, including a semantic control network (Noonan et al., 2013; Hoffman et al., 2015; Mineroff et al., 2018; Cotosck et al., 2021; Gao et al., 2021; Hodgson et al., 2021; Smith et al., 2021).

### Semantic connectivity at advanced age

The findings of the current study do not suggest an obvious or marked decline in the neural response of the semantic system among older adults. Within the sample of middle aged to oldest-old adults, greater age was not associated with major alterations in functional connectivity among the study participants, though a few age-related effects were evident. Greater age was associated with increased connectivity between the left AG and the proximal cortical areas of the supramarginal gyrus for overall semantic and also concrete word processing compared to rhyme word processing, suggesting greater engagement of association areas adjacent to the AG hub. With greater age, adults exhibited strengthening of functional connections to the supramarginal gyrus, an area proximal to the AG. That age-associated differences in the extent or strength of connectivity with more distal cortical areas and networks were not evident is noteworthy and suggests that between the ages of 55 and 85 functional connectivity of semantic systems of the cortex are relatively well preserved (Eustache et al., 1998; Vaughan et al., 2016). During this period of the lifespan, declines are common across many other fluid cognitive functions (Salthouse, 1976; Salthouse et al., 2000; O’Shea et al., 2018), including verbal fluency, and word and name retrieval (Albert et al., 1988; Brickman et al., 2005; Vaughan et al., 2016). Some recent studies have reported age-associated changes in semantic networks (Hoffman and Morcom, 2018; Martin et al., 2022). However, these effects tended to occur in frontal cortical areas on tasks with high demand for semantic control, and often were evident with respect to differences in the intensity of activation rather than connectivity. Also, these studies tended to compare old vs. young adults across a wide age range. Given the lack of a young cohort in the current study, conclusions cannot be made about possible age-associated differences in functional connectivity across the lifespan, or between young versus older adults. Yet, the current findings support the relative stability of functional connectivity during semantic processing among adults between the ages of 55 and 85, a period of life when cognitive decline is of particular concern.

The overall characteristics of connectivity in this cohort align with those reported for similar tasks in young adults. Since a younger adult sample was not included in the current study for direct comparison of older and younger adults, the integrity of semantic hubs throughout the entire lifespan cannot be commented within the scope of the paper; however, within middle aged to older adults, the general connectivity of the core semantic regions was not significantly altered as a function of age. This idea that the core regions of the semantic network remain intact while the ancillary “spokes” or linguistic-executive components change in healthy aging does have some support. A recent study utilizing a living vs. non-living semantic judgment task demonstrated that, in response to an effortful semantic demand, older adults activate the contralateral frontal and parietal cortices, regions of the default mode network, and bilateral superior parietal cortices (Badre et al., 2005). These results correspond with our general hypothesis that older adults may demonstrate a more diffuse semantic system, though the authors do not specify whether the additional activation is truly compensatory or if it represents a decrease in the inhibition of irrelevant structures. They also demonstrated that older adults do not modulate activations of general frontal-parietal control systems in response to semantic task demand. These results loosely correspond to our finding that the connectivity of the frontal-parietal control system and semantic hubs was reduced in our sample compared to those reported previously. Thus, when contextualized within the body of work examining young adults, the current study contributes to preliminary results in the literature suggesting that the functioning of the hubs of the semantic system is largely age invariant. However, older adults may utilize a less cohesive network, including contralateral perisylvian cortex and linguistic executive regions, and are less capable of appropriating multidomain systems to respond to increased semantic task demands. Contextualized within this approach to linguistic functioning and age, the lack of seed-based changes to the functional connectivity of the semantic system is sensible. Rather, aging may differentially affect overall network or sub-network measures of coherence, which are not readily computed during the seed-to-voxel type of connectivity analysis.

### Study limitations

Several factors may limit the current study’s generalizability. The study did not directly compare older and younger adults, nor did it longitudinally compare adults over time. To specifically probe how these results change over the lifespan, future investigations of association and semantics should include a cohort of younger adults (i.e., aged 20–50 years). Doing so would enable a comparison of the semantic functioning at advanced age compared to age effects across the lifespan. Secondly, this study included a relatively small sample size (*n* = 38), which may have further limited sensitivity to aging effects. Future studies may wish to expand both the age range and the number of participants to capture potential subtle aging effects. Investigators should also consider utilizing network-based approaches to functional connectivity to characterize network coherence rather than relying on seed-regions. They may also wish to include regions that are theoretically unrelated to semantic functioning, as potential control regions. Unfortunately, we were not able to employ a multi-echo scanning protocol during the acquisition of BOLD fMRI, though such protocols can enhance signal in cortical areas vulnerable to artifact. Activation in the anterior temporal cortex, including the temporal poles, was evident across task conditions, suggesting that there had not been dramatic signal loss from this area. However, some loss of signal strength may have occurred and reduced effect sizes for connectivity with this region. Finally, seed-to-voxel based analyses may have been influenced by the relatively large anatomically defined seeds. Recent literature suggests, for example, that different regions of the angular gyrus are important for default mode versus semantic processing (Badre et al., 2005; Seghier et al., 2010; Seghier, 2013). Future studies should segregate key regions of the semantic system to allow for detection of such differences.

## Conclusion

Older adults exhibit robust connectivity between aMTG, AG, and IFG and multiple bilateral cortical areas during semantic processing. Differences in the strength and extent of connectivity from these putative semantic hubs (aMTG, AG) and the IFG semantic control area exist as a function of demand for abstract versus concrete semantic processing. Between the ages of 55 and 85 years, functional connectivity was relatively stable during abstract and concrete processing. Semantic processing elicited greater connectivity between only left AG and left supramarginal gyrus as a function of more advanced age, only during concrete semantic processing. Significant degradation of functional connectivity during semantic processing between the ages of 55 and 85 years was not evident. As the study focused on semantic functioning in older adults, young adults were not assessed which may limit generalizability. Future longitudinal task-based fMRI studies comparing the functional connectivity of young and older adults on tasks differing in semantic demands will be valuable.

## Data availability statement

The raw data supporting the conclusions of this article will be made available by the authors, following a review by the corresponding author of the intended use of the data.

## Ethics statement

The studies involving human participants were reviewed and approved by Internal Review Board (University of Florida). The patients/participants provided their written informed consent to participate in this study.

## Author contributions

RC contributed as corresponding author and worked with AG on all aspects of the study, study design, fMRI acquisition, analyses, interpretation of results, and manuscript writing. AG contributed as lead author, took the lead on acquisition and analyses. AG and RC shared primary responsibility for interpretation of results and manuscript writing. EP played a major role in helping AG with paradigm implementation with eprime, analysis, and manuscript preparation. AW contributed to study design and manuscript preparation and editing. JW contributed through literature review, sections of the introduction, interpretation of results, and editing of final manuscript. All authors contributed to the article and approved the submitted version.

## Footnotes

1
https://www.fil.ion.ucl.ac.uk/spm/
